# CBD2: A functional biomarker database for colorectal cancer

**DOI:** 10.1002/imt2.155

**Published:** 2023-12-04

**Authors:** Xueli Zhang, Min Li, Siting Ye, Ke Shen, Haining Yuan, Shoaib Bakhtyar, Qiliang Peng, Yongsheng Liu, Yingying Wang, Manshi Li, Chi Zhang, Yixin Wang, Xiaohe Bai, Shunming Liu, Ke Zhao, Bairong Shen, Dirk Repsilber, Guang Hu, Hong Zhang, Xiao‐Feng Sun

**Affiliations:** ^1^ Medical Research Institute, Guangdong Provincial People's Hospital (Guangdong Academy of Medical Sciences) Southern Medical University Guangzhou China; ^2^ Department of Oncology and Department of Biomedical and Clinical Sciences Linköping University Linköping Sweden; ^3^ Department of Ophthalmology, Guangdong Provincial People's Hospital (Guangdong Academy of Medical Sciences), Guangdong Eye Institute Southern Medical University Guangzhou China; ^4^ Guangdong Provincial Key Laboratory of Artificial Intelligence in Medical Image Analysis and Application Guangzhou China; ^5^ MOE Key Laboratory of Geriatric Diseases and Immunology, Suzhou Key Laboratory of Pathogen Bioscience and Anti‐infective Medicine, Department of Bioinformatics, Center for Systems Biology, School of Biology and Basic Medical Sciences Suzhou Medical College of Soochow University Suzhou China; ^6^ Department of Ultrasound The Second Affiliated Hospital of Guangzhou University of Chinese Medicine Guangzhou China; ^7^ Department of Orthopaedics The Second Affiliated Hospital of Guangzhou University of Chinese Medicine Guangzhou China; ^8^ Department of Critical Care Medicine and Institutes for Systems Genetics, Frontiers Science Center for Disease‐related Molecular Network, West China Hospital Sichuan University Chengdu China; ^9^ School of Laboratory Medicine and Bioengineering Hangzhou Medical College Hangzhou China; ^10^ School of Medicine, Institute of Medical Sciences Örebro University Örebro Sweden; ^11^ Department of Radiotherapy and Oncology The Second Affiliated Hospital of Soochow University Suzhou China; ^12^ Department of Immunology, Genetics and Pathology Uppsala University Uppsala Sweden; ^13^ Key Laboratory of Public Health Safety, School of Public Health Fudan University Shanghai China; ^14^ Department of Otolaryngology Guangzhou Women and Children's Medical Centre Guangzhou China; ^15^ School of Medicine The Chinese University of Hong Kong, Shenzhen Shenzhen China; ^16^ Department of Mathematics University of California San Diego California USA; ^17^ Department of Radiology, Guangdong Provincial People's Hospital Guangdong Academy of Medical Sciences Guangzhou China; ^18^ Institutes for Systems Genetics, Frontiers Science Center for Disease‐Related Molecular Network, West China Hospital Sichuan University Chengdu China

**Keywords:** biomarker, colorectal cancer, database, network analysis

## Abstract

The rapidly evolving landscape of biomarkers for colorectal cancer (CRC) necessitates an integrative, updated repository. In response, we constructed the Colorectal Cancer Biomarker Database (CBD), which collected and displayed the curated biomedicine information for 870 CRC biomarkers in the previous study. Building on CBD, we have now developed CBD2, which includes information on 1569 newly reported biomarkers derived from different biological sources (DNA, RNA, protein, and others) and clinical applications (diagnosis, treatment, and prognosis). CBD2 also incorporates information on nonbiomarkers that have been identified as unsuitable for use as biomarkers in CRC. A key new feature of CBD2 is its network analysis function, by which users can investigate the visible and topological network between biomarkers and identify their relevant pathways. CBD2 also allows users to query a series of chemicals, drug combinations, or multiple targets, to enable multidrug, multitarget, multipathway analyses, toward facilitating the design of polypharmacological treatments for CRC. CBD2 is freely available at http://www.eyeseeworld.com/cbd.

## Highlights

- CBD2 integrates biomarkers derived from diverse biological sources and clinical applications, employing a standardized approach for annotation and classification.
- CBD2 facilitates precision medicine in colorectal cancer by providing abundant data, including drug–target information and visualized network analysis functions.
- With its user‐friendly interface, CBD2 aids in the identification and development of biomarkers for colorectal cancer.

## Graphical abstract

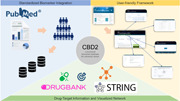

## INTRODUCTION

Colorectal cancer (CRC) remains a major global challenge in terms of its diagnosis, treatment, and prognosis, despite being the third most prevalent cancer worldwide [1]. Biomarkers have been demonstrated to enhance the clinical effectiveness of CRC, and their discovery is a growing area of research, as evidenced by the publication of over 100,000 papers on PubMed. However, sifting through this vast amount of literature is time‐consuming and may lead to loss of accuracy. To provide researchers with a comprehensive platform to search for accurate and categoric information for CRC biomarkers, we published the Colorectal Cancer Biomarker Database (CBD) in 2018, which gathered the curated data for all the reported 870 biomarkers for CRC [2]. Biomarkers in CBD are classified based on their biological sources into DNA, RNA, protein, and others. As a supplement to CBD, the Epigenetic Biomarker Database for Colorectal Cancer was developed in 2020 to collate epigenetic biomarkers for CRC [3]. Recently, some relevant biomarker databases have been reported, such as the MarkerDB [4] and the Cancer Biomarkers database [5]. However, the biomarkers for CRC are significantly lower than our CBD. With biomarkers from additional sources such as metabolite, microbiome, and image being reported in recent years, there is a growing need for continued development and expansion of biomarker databases for CRC.

Although identifying objects that are not viable biomarkers for CRC, as highlighted by negative experimental results, is a crucial aspect of biomarker studies, this aspect has received inadequate attention. To help researchers avoid such pitfalls and enhance the efficacy of biomarker discovery, a database specifically dedicated to the collection and dissemination of nonbiomarker information would be of great benefit.

Protein is the primary driver of the major physiological processes in the human body, and it constitutes the primary component of CRC biomarkers. Protein–protein interaction (PPI) networks facilitate the function of proteins by enabling their interaction with each other. In our previous study, we found that the biomarker–biomarker interaction (BBI) network exhibited distinct network features. However, specific BBI networks for specific groups of CRC biomarkers have not been investigated.

**Figure 1:**
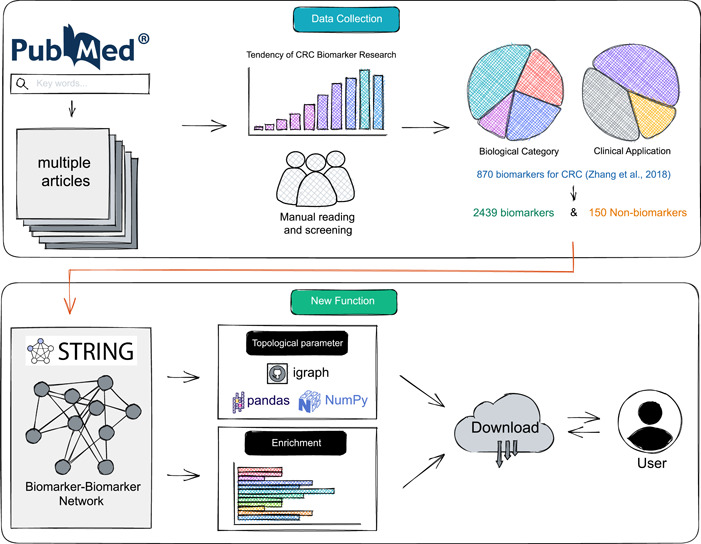
The pipeline of CBD2. We manually collected and classified validated biomarkers and nonbiomarkers by manually reading the literature, and added innovative network construction and analysis functions. CBD, Colorectal Cancer Biomarker Database.

In this study, we present the CBD2 database (http://www.eyeseeworld.com/cbd), including a substantial number of newly discovered biomarkers, as well as nonbiomarkers, and incorporate network visualization and topology analysis functions. By introducing these features, CBD2 represents a user‐friendly and multifunctional platform that can aid in the discovery and development of CRC biomarkers. The overall pipeline of CBD2 is illustrated in the diagram (Figure 1).

## DATABASE DESCRIPTION

In this work, we describe an updated version of the comprehensive CBD platform for CRC biomarkers, first proposed in 2018. The updated version, named CBD2, includes more detailed information on 1569 newly validated biomarkers and 150 nonbiomarkers. An innovative feature of CBD2 is the newly developed “Explore” page that combines the functions of the STRING API and igraph package. This feature enables users to easily query the interaction network of the biomarkers of interest. Further, the hyperlinks of functional enrichment analysis results are provided. All of these results are freely available for download.

### Data summary

Compared with CBD, CBD2 updates 1569 biomarkers, bringing the total number of biomarkers to 2439. CBD2 expands the biological categories of biomarkers to DNA, RNA, protein, metabolite, microbiome, and image, as well as the clinical categories of diagnosis, treatment, and prognosis. Furthermore, CBD2 includes 150 nonbiomarkers, which refer to objects that have been deemed unsuitable for serving as CRC biomarkers. Notably, all biomarkers are annotated based on the DrugBank database, we provide information on known drug targets and corresponding drug molecules. A comparison of the data contained in CBD2 and CBD is conducted (Table 1). All the data included in CBD2 could be downloaded via the “Download” page.

**Table 1:** Data comparison between CBD2 and CBD.

	CBD	CBD2
Year range	1986–2017	2018–Dec 2022
Number of articles	1115	2391 (+1276)
Number of biomarkers	870	2439 (+1569)
Number of nonbiomarkers	\	150
STRING network	\	1271
Drug–target data	\	520 known targets
		4883 drugs

Abbreviation: CBD, Colorectal Cancer Biomarker Database.

### Function

**Figure 2:**
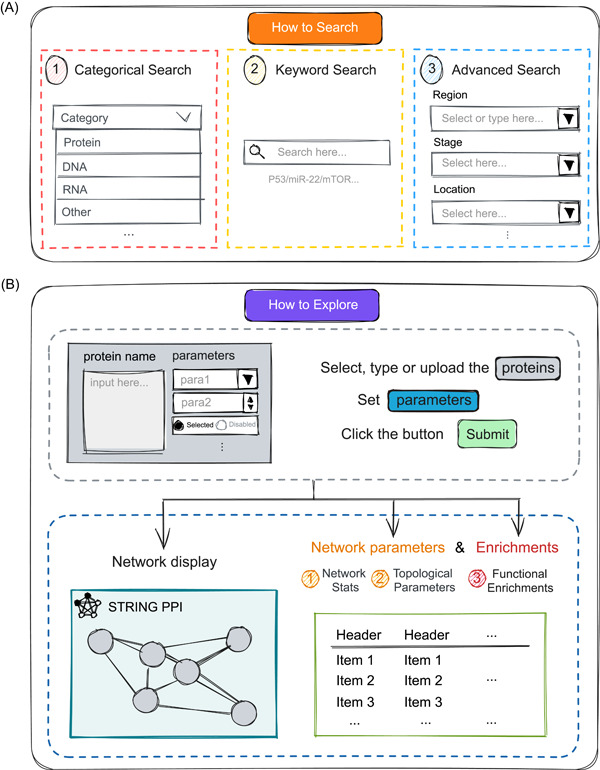
Simple guide on how to use the functions available in CBD2. (A) Three different ways offered by us to retrieve data: Categorical search, Keyword search, and Advanced search. (B) Steps and schematic diagram of the network analysis function module. CBD, Colorectal Cancer Biomarker Database.

CBD2 presents a user‐friendly and multifunctional framework (Figure 2). Users can search for biomarker information on the “Biomarkers” page via categorical search, keyword search, or advanced search. After submitting a search query, a detail page for the specific biomarker is provided, encompassing detailed biomedicine information such as biological categories, ontology‐based descriptions, and a link to the relevant page of the NCBI database. Patient information, including the region, race, gender, and age of patients in the source study, as well as cancer information such as the location and stage of CRC, and experimental information such as the method, statistical results, application, conclusion, author, journal, published year, and PMID are also available. The information pertaining to nonbiomarkers is not categorized in detail and can be accessed on the “Non‐Biomarkers” page.

CBD2 incorporates drug–target information provided by DrugBank (v5.1.10) [6], enabling users to determine whether their searched biomarker is a drug target. On the detailed information page, users can access the PPI network for a specific protein biomarker by clicking the relevant button. The network is obtained from the STRING database [7], allowing users to manipulate and access structure and biomedicine information for each node protein. Additionally, some biomarker details may include annotated supplementary content.

CBD2 includes PPI information for all CRC protein biomarkers via the functions of the STRING API [7]. Users have the capability to investigate BBI networks by adhering to the parameter‐setting guidelines delineated on the “Explore” page. Additionally, a comprehensive elucidation of the implications of these parameters is furnished (Table S1). CBD2 provides three ways to query the BBI network: (1) directly selecting a biomarker stored in CBD2; (2) uploading a Txt or CSV file with one protein per line; (3) directly typing in a multiline text box. Then, the operational and visual BBI network will appear on the webpage.

Meanwhile, CBD2 employs the igraph package [8] to calculate network topological features of the BBI network and specific nodes, such as Degree, Betweenness, and Closeness. Biological functional analyses such as pathway enrichment analysis, gene ontology (GO) annotations, tissue expression, disease–gene association, annotated keywords in UniProt, protein domains and features, reference publications in PubMed, human phenotype from Monarch, and local network cluster are provided.

Users are welcome to submit the newly reported biomarkers on the “Submission” page.

## CASE STUDY 1: INTEGRATED NETWORK ANALYSIS OF BIOMARKERS IN CBD2

To showcase the clinical relevance of the identified CRC biomarkers for diagnosis, treatment, and prognosis, we presented a case study that demonstrates the utility of CBD2 for analyzing biomarkers in a practical setting. We constructed the BBI networks for the existing diagnostic, therapeutic, and prognostic protein biomarkers in CBD2 (Figure S1) and calculated network parameters in the “Explore” page, followed by GO functional enrichment analysis and Kyoto Encyclopedia of Genes and Genomes (KEGG) pathway enrichment analysis (Figure S2).

We found that the number of biomarkers used for the prognosis of CRC is significantly higher than those used for diagnosis and treatment, which is consistent with medical practice: (1) Various factors, such as tumor size, location, degree of differentiation, metastasis, and treatment options, could impact the survival period and quality of life of cancer patients after treatment. Therefore, multiple biomarkers were needed to comprehensively assess the prognosis. (2) Specific and precise biomarkers were needed to guide the determination of cancer type and stage and the selection of appropriate treatment methods. Therefore, diagnosis and treatment may require only a few biomarkers. What's more, early diagnosis and effective treatment of cancer remained significant challenges, which was also a crucial factor contributing to the relatively small number of biomarkers available for these purposes.

**Figure 3:**
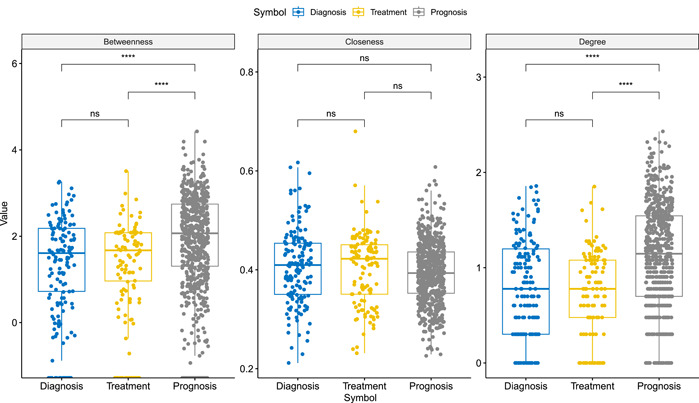
Box plot of topological parameters for existing diagnostic, therapeutic, and prognostic protein biomarkers in CBD2. Due to the large differences in the respective groups for Betweenness and Degree, we performed a log transformation on these two parameters. Comparisons labeled as “ns” denote “not significant,” indicating that the statistical tests did not find a significant difference between the compared groups. CBD, Colorectal Cancer Biomarker Database.

According to the boxplot of topological parameters (Figure 3), we noticed that the Closeness parameter had no obvious intergroup difference, while for Betweenness and Degree, the difference mainly appears in the Prognosis group and the other two marker groups.

The degree in PPI networks refers to the number of connections that a given protein has with other proteins, indicating its level of connectivity. Betweenness was a measure of the number of shortest paths that pass through a given protein, indicating its importance as a mediator of information flow within the network. Closeness measures the average shortest path between a protein and all other proteins in the network. It reflected how easily information can be transmitted from one protein to another in the network.

It is noteworthy that the scale of the network might influence these metrics, especially Degree. Larger networks might have a higher average degree, but this also depends on the sparsity or density of the network. For Betweenness, more “bridging” nodes might exist in larger networks, but this is not always the case since it depends on the specific structure of the network.

Considering the above situation, the following conclusions were indicated: (1) the lack of intergroup difference in the Closeness parameter suggested that the nodes in the different marker groups were similarly connected and can transmit information efficiently in the network. This may indicate that the selected biomarkers for CRC diagnosis, treatment, and prognosis were all functionally related and contributed to the overall network connectivity; (2) the differences in the Betweenness and Degree parameters between the prognosis group and the other two marker groups indicated that the nodes in the prognosis group have a greater influence on the network structure than the nodes in the diagnosis and treatment groups. This may imply that the selected biomarkers for CRC prognosis are more important for the overall network structure and function.

We performed an enrichment analysis of the resulting proteins. The obvious difference between the cellular components enriched for biomarkers for the diagnosis, treatment, and prognosis of CRC was that they reflect different aspects of the disease. The cellular components enriched for biomarkers for the diagnosis of CRC, such as the collagen‐containing extracellular matrix and platelet alpha granule, were primarily involved in the regulation of cell proliferation, migration, and invasion, which are important processes in cancer development and progression. The cellular components enriched for biomarkers for treatment of CRC, such as focal adhesion and cell–substrate junction, were involved in the regulation of cell adhesion, migration, and signaling, which were important processes in drug response and resistance. The cellular components enriched for biomarkers for CRC prognosis, such as secretory granule lumen and collagen‐containing extracellular matrix, were involved in the regulation of cell survival, proliferation, and metastasis, which were important processes in cancer recurrence and survival.

Analogously, comparable outcomes could be inferred regarding the molecular functions and biological processes of these biomarkers. However, we placed greater emphasis on the pathway enrichment of these biomarkers rather than on functional enrichment data. From the perspective of their respective enriched pathways, we found that there were highly overlapping co‐enriched pathways among biomarkers of different aspects of CRC. For example, “Proteoglycans in cancer” pathway was enriched in biomarkers for the diagnosis, treatment, and prognosis of CRC, suggesting that this pathway may play a key role in all stages of CRC. Likewise, the “PI3K‐Akt signaling pathway” was enriched in biomarkers for the diagnosis, treatment, and prognosis of CRC, while the pathway “EGFR tyrosine kinase inhibitor resistance” was enriched in biomarkers for the treatment and prognosis of CRC. These overlaps in enriched pathways suggests that some pathways may have multifaceted roles in CRC, and targeting them may have broader implications for the diagnosis, treatment, and prognosis of CRC. The network construction and analysis results of the above three types of biomarkers could be obtained with one click on our “Explore” page.

## CASE STUDY 2: COMPARISON OF THE SAME BIOMARKER IN DIFFERENT STUDIES

Several biomarkers have undergone extensive investigations across multiple research endeavors, all of which have been meticulously curated and incorporated into the comprehensive CBD2 database. The investigation of biomarkers across multiple studies presents an intriguing avenue for exploration. As such, a comparative analysis of the impact of a specific biomarker within distinct research investigations holds substantial scientific merit.

Take *p53* into consideration. In the realm of cellular biology, the *p53* protein holds paramount importance in ensuring genomic stability. Under normal circumstances, this tumor suppressor protein acts as a gatekeeper, halting the progression of defective cells and directing them toward apoptosis or cellular senescence. This inherent ability of *p53* to maintain cellular equilibrium is undermined when mutations or functional anomalies intervene. Mutated or aberrantly activated *p53* often results in compromised tumor‐suppressive functions, allowing damaged cells to proliferate unchecked, subsequently amplifying the risk of tumorigenesis.

We searched CBD2 for collected *p53*‐related information and found that almost all the research related to it focused on prognosis. A summary of the research contents from six studies is presented (Table 2) [9, 10, 11, 12, 13, 14]. Across these investigations, a consistent observation was evident: patients with *p53*‐positive expression consistently faced a higher recurrence rate in comparison to those with *p53*‐negative status. This trend invariably translated to a notably reduced survival rate for the latter group.

**Table 2:** Research contents of six literatures for *p53*.

*p53*		Patients number		Recurrence rate		Median survival (months)		2‐Year survival rate		3‐Year survival rate		5‐Year survival rate	
ID	PMID	*p53* positive	*p53* negative	*p53* positive	*p53* negative	*p53* positive	*p53* negative	*p53* positive	*p53* negative	*p53* positive	*p53* negative	*p53* positive	*p53* negative
1	1451055	39	61	23.8	5.9	\	\	\	\	61.8	96.7	\	\
19	7931472	50	57	60	35	\	\	\	\	\	\	48	78.9
31	8347496	121	82	Higher	Lower	\	\	\	\	\	\	58.1	76.3
42	8874329	72%	28%	\	\	21	53.2	41.7	78.6	\	\	\	\
78	9797697	44	25	\	\	27	93	\	\	31.5	71.8	21	53.1
202	12818291	38	37	\	\	\		\		\	\	63.2	86.5

## CASE STUDY 3: NCRNA–GENE INTERACTION NETWORK

The elucidation of noncoding RNA (ncRNA)–gene interaction networks holds paramount significance in contemporary biomedical research. These intricate networks provide invaluable insights into the regulatory mechanisms governing gene expression and cellular processes. By unraveling the complex interplay between ncRNAs and genes, we gain a deeper understanding of their functional roles in physiological and pathological contexts. This knowledge has far‐reaching implications, ranging from the identification of novel therapeutic targets to the development of innovative diagnostic and prognostic tools. The exploration of ncRNA–gene interaction networks thus represents a crucial endeavor in advancing our understanding of molecular biology and its implications for human health.

Three and seventy‐four ncRNA biomarkers have been collected in CBD2, of which 210 belong to microRNA (miRNA). We finally parsed 251 standard miRNA identifiers from them. By constraining the Organism parameter to “H. sapiens (human)” and refining the Tissue parameter to “Intestine”, these miRNAs, in conjunction with 796 protein biomarkers from CBD2, were input into miRNet [15] with the aim of constructing a network elucidating the interactions between ncRNA and genes.

**Figure 4:**
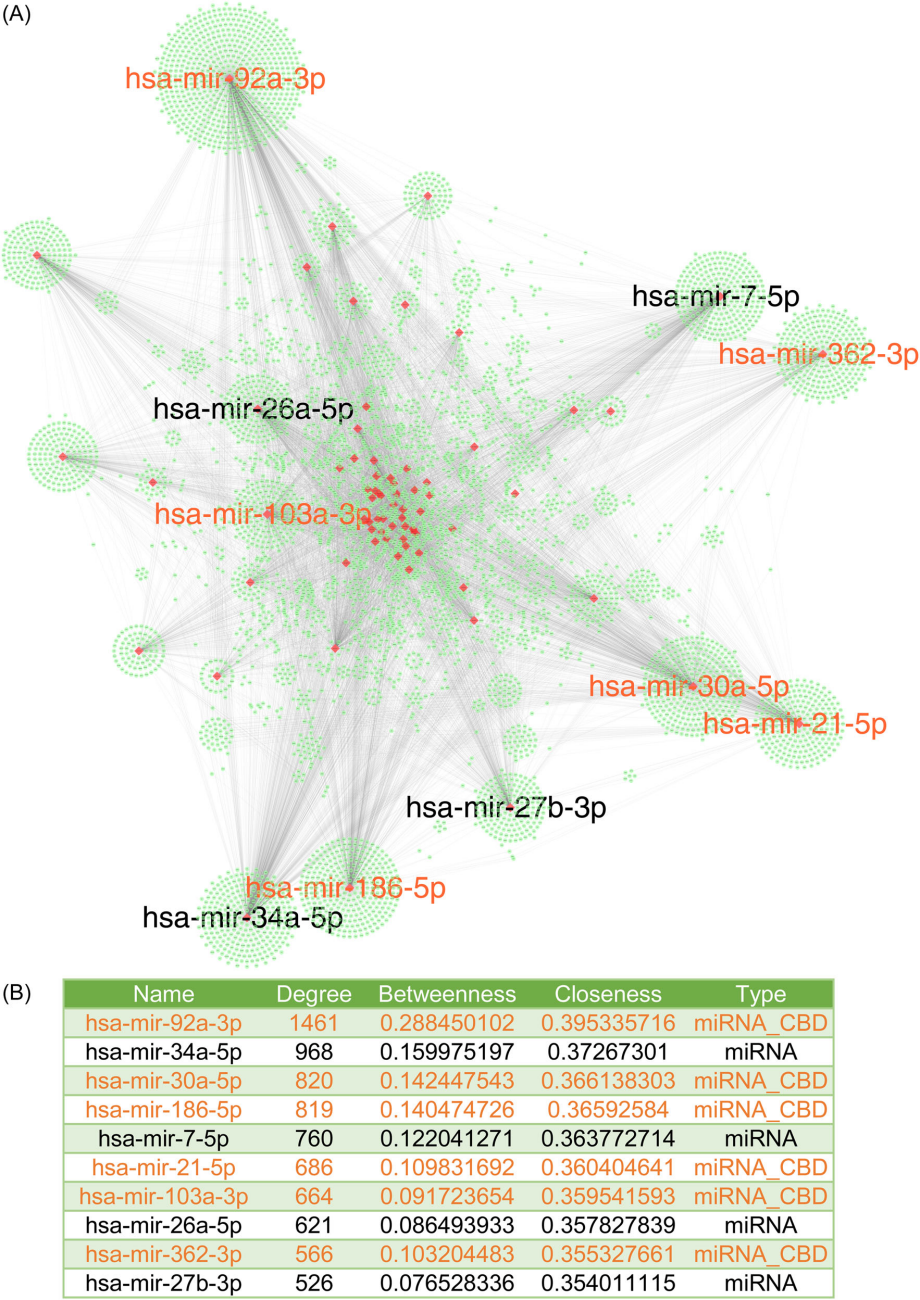
ncRNA–gene interaction network of the biomarkers in CBD2. Following the analytical procedure through miRNet, an interactive subset consisting of 713 proteins (genes) and 15 miRNAs was identified from the initial input. (A) The derived network encompasses a total of 6235 genes and 72 miRNAs, forming a complex structure with 13,670 edges. The green nodes represent genes, and the red nodes represent miRNAs. The labels for the miRNAs that rank in the top 10 in terms of degree among all miRNAs are displayed. (B) Basic topological parameter information of the top 10 miRNAs in terms of degree. The miRNA collected in CBD2 is highlighted in orange. CBD, Colorectal Cancer Biomarker Database; miRNA, microRNA; ncRNA, noncoding RNA.

In the ncRNA–gene interaction network (Figure 4). We found that *hsa‐mir‐92a‐3p*, *hsa‐mir‐103a‐3p*, *hsa‐mir‐21‐5p*, *hsa‐mir‐7‐5p*, *hsa‐mir‐34a‐5p*, *hsa‐mir‐30a‐5p*, *hsa‐mir‐26a‐5p*, *hsa‐mir‐186‐5p*, and *hsa‐mir‐362‐3p* were the hubs in the network, seven of them are included in CBD2. We additionally conducted enrichment analysis utilizing both KEGG and DisGeNET on the delineated network, the results of which exhibited a pronounced correlation with CRC, underscoring the potential significance of the identified ncRNA–gene interactions in the context of this malignancy.

The files generated during the network construction process and relevant details are provided in the supplementary tables (Tables S2 **–** S5).

## DISCUSSION

In this study, we have updated the CRC biomarker database (CBD2), which has included 2439 reported biomarkers for CRC. We have also added the BBI network analysis function to this update. We anticipate that the updated version of CBD2 will serve as a flexible and valuable tool for advancing CRC biomarker research, particularly with the addition of powerful network construction and analysis capabilities.

Except with our CBD2 database, other biomarker databases have been reported, such as the Global Online Biomarker Database, which amasses a plethora of benchmark biomarkers (https://gobiomdb.com/). Another significant repository is the Early Detection Research Network established by the US National Cancer Institute (https://edrn.nci.nih.gov/). Databases with more specific pathophysiological focuses include the US Environmental Protection Agency Biomarker Database catering to children's healthcare (https://cfpub.epa.gov/ncea/risk/recordisplay.cfm?deid=85844) [16], the Tuberculosis Biomarker Database specifically for tuberculosis (https://www.finddx.org/publication/tuberculosis-biomarker-database-2/) [17], and the Infectious Disease Biomarker Database dedicated to infectious diseases (http://biomarker.cdc.go.kr) [18]. Furthermore, for oncology‐centric research, there is the Gastric Cancer (Biomarkers) Knowledgebase for gastric cancer (http://biomarkers.bii.a-star.edu.sg/background/gastricCancerBiomarkersKb.php) [19], and the Liver Cancer Biomarker Reference Into Function (LiverCancerMarkerRIF) tailored for liver cancer (http://btm.tmu.edu.tw/LiverCancerMarkerRIF/) [20]. An eye biomarker database released by us provided a standardized platform for ocular biomarkers, and a driving force for future ophthalmic precision medicine [21]. These platforms offer invaluable insights for both the research community and clinical practitioners. Comparing with these databases, the CBD2 holds significant advantages:

1. *Comprehensive focus on CRC*: While many databases diversify their biomarker collection across multiple diseases, the CBD2 specifically concentrates on CRC, offering a depth of information unparalleled by more generalized repositories.
2. *Advanced analytical tools*: CBD2 is not just a data repository; it offers advanced analytical tools that enable users to conduct preliminary analyses. For example, we added network analysis capabilities to the database for the first time, providing users with new perspectives on the interaction level of biomarkers.
3. *Rigorous quality control*: Each entry in the CBD2 undergoes a rigorous review process, ensuring that the data is not only comprehensive but also of high quality, minimizing the risk of errors or inaccuracies.
4. *Extensive metadata inclusion*: CBD2 does not just provide biomarker data. It offers extensive metadata, giving researchers context about experimental conditions, patient demographics, and other relevant parameters.
5. *Open access*: Ensuring equitable access to knowledge, CBD2 operates on an open‐access model. Not only is its wealth of information freely available to researchers, clinicians, and the public at large without any barriers, but our website code and data tables are also openly available.

In future versions, we are planning to replace most of the manual reading work with methods based on natural language processing for biomedical text mining, retaining only a subset of experts for quality control. This will make the inclusion, exclusion, and information extraction of subsequent literature more efficient. We plan to update this database every 1–2 years.

In summary, the advancement of precision medicine in the field of CRC becomes more possible through the rich and expanded information provided by CBD2.

## METHODS

### Data collection and management

The literature search was performed on PubMed up to December 2022. We found 1276 new papers related to CRC biomarkers, and CBD2 currently contains information on CRC biomarkers from a total of 2391 papers. A collection of these papers is available on the CBD2's download page.

We selected papers that met the following criteria:

The study explicitly indicates that the object of study could be utilized as any biomarker for human CRC.

1. The study conducted experiments with control groups and demographic characteristics to validate their conclusions.
2. The experimental design and methods are described in detail in the paper.
3. Analyses conducted in the study ought to yield statistically significant outcomes. For instance, treatment or prognosis biomarkers must exhibit a *p* value for odds ratio, hazard ratio, or relative risk that is less than 0.05, underscoring the robustness of the findings.
4. The sample size in the study should be greater than 30.

In our review of selected articles, we meticulously extracted both biomarker‐specific details (such as the biomarker name, biological category, and description) and experimental information (inclusive of intricate details such as geographic region, racial background, sample size, gender, age, source, experimental method, statistical analysis, applications, conclusions), and article specifics (like first author, publishing journal, year of publication, and PubMed ID). The description of the collected information has been presented (Table 3).

**Table 3:** Descriptions for the collected information in CBD2.

Items	Description
ID	Biomarker ID in the database
Name	Name of (non) biomarker
Category	Biological type of biomarker (e.g., protein, DNA)
NCBI protein	Protein information in the National Center for Biotechnology Information (NCBI)
Description	Description of biomarker
Region	The region where this biomarker research from
Race	The sample race of this biomarker research
Number	Sample number included in this biomarker research
Gender (male/female)	Gender distribution of sample number: Male/female
Age	Ages of samples: Mean age (minimum–maximum)
Location	Cancer location
Stage	Cancer stage
Source	Sample source
Experiment	Experiment method for research
Statistics	Statistics result for a biomarker in research
Application	Biomarker application
Clinical use	Whether the biomarker has been used in clinical practice
Conclusion	Research conclusion for this biomarker
Reference	First author, published Journal, and year of research
PMID	PubMed ID of research
STRING name	Name of the protein in the STRING database (If available)
STRING PPI	Show the protein–protein interaction (PPI) network of this protein with Other proteins with top 10 confidence scores (If STRING name is available)
Know target	Whether the current biomarker is a documented target in DrugBank
Drugs	Drugs on the target that have been recorded in DrugBank (if any)
Addition	Note by us

To further ensure the reliability of our data, we assessed the quality of each selected paper. Utilizing the Critical Appraisal Skills Program (CASP, https://casp-uk.net/casp-tools-checklists/) checklists, we determined the confidence score of the papers. These checklists pose 11 questions designed to gauge the quality of a study's design, methodology, and results. For the majority of these questions, evaluators would respond with “Yes,” “No,” or “Can't tell” reflecting the paper's quality. A paper with answers comprising nine or more “Yes” would be regarded as of high quality. Those with answers between six and eight would be deemed of medium quality, while those with fewer than six “Yes” responses would be categorized as low quality. Studies that failed to meet more than five CASP criteria were excluded from our database.

### Data cleaning

Standardization of geographic region names was achieved using the API provided by the GeoNames geographic database (https://www.geonames.org/). Further, for protein biomarkers, we accessed the corresponding STRING IDs and symbols from the STRING database (https://string-db.org/). Regarding the potential of a biomarker to serve as a drug target, we extracted pertinent data from DrugBank (RRID:SCR_002700) (https://go.drugbank.com/) for mapping purposes. In cases where the biomarker is identified as a known drug target, we also furnished comprehensive drug information pertaining to the biomarker from DrugBank.

### Data analysis

We conducted a thorough extraction of protein and gene biomarkers specific to CRC, subsequently mapping these onto the human protein interaction network to engineer a BBI network. During the development of this network, certain topological characteristics were employed to depict the interconnectivity within the network. The realization of this functional component was achieved via the utilization of the STRING API and the Python package, igraph.

GO functional enrichment analysis and KEGG pathway enrichment analysis were conducted by the R package clusterProfiler [22].

### Tools and software

CBD2 operates within a WNMP (Windows Server, Nginx, MySQL, PHP) environment hosted on a cloud server. The web interface is developed using a combination of HTML, PHP, and JavaScript technologies. The network analysis interface, on the other hand, is facilitated through the application of gradio (https://www.gradio.app/).

## AUTHOR CONTRIBUTIONS

Xueli Zhang contributed to conceptualization, methodology, software, investigation, supervision, project administration, and writing—review and editing. Min Li contributed to software, data curation, formal analysis, visualization, project administration, and writing—original draft. Siting Ye contributed to data curation, formal analysis, visualization, and writing—review and editing. Ke Shen and Haining Yuan contributed to conceptualization, methodology, formal analysis, investigation, and data collection. Shoaib Bakhtyar, Qiliang Peng, Yongsheng Liu, Yingying Wang, Manshi Li, hi Zhang, Xiaohe Bai, and Ke Zhao contributed to resources, data collection, data curation, and formal analysis. Yixin Wang contributed to resources, software, data curation, and formal analysis. Shunming Liu contributed to methodology, data collection, data curation, and formal analysis. Bairong Shen and Dirk Repsilber contributed to conceptualization, methodology, and data curation. Guang Hu, Hong Zhang, and Xiao‐Feng Sun contributed to conceptualization, supervision, project administration, and writing—review and editing.

## CONFLICT OF INTEREST STATEMENT

The authors declare no conflict of interest.

## Supporting information

Supporting Information

Supporting Information

**Figure S1:** BBI networks for the existing diagnostic, therapeutic, and prognostic protein biomarkers in CBD2.
**Figure S2:** Functional analysis results for biomarkers included in CBD2.

**Table S1:** The meaning of the parameters used to extract protein‐protein interactions from the STRING database.
**Table S2:** Gene‐to‐miRNA Interaction Table.
**Table S3:** miRNA‐to‐Gene Interaction Table.
**Table S4:** Results of KEGG enrichment analysis on the miRNA‐gene network.
**Table S5:** Results of DisGeNET enrichment analysis on the miRNA‐gene network.

## Data Availability

The data sets generated and analyzed during the study are available for download at: http://www.eyeseeworld.com/cbd/Download.html. The code used to build our database in this study is open source and available on GitHub at: https://github.com/WhyLIM/CBD. Supplementary materials (figures, tables, scripts, graphical abstract, slides, videos, Chinese translated version and update materials) may be found in the online DOI or iMeta Science http://www.imeta.science/.
