# Supplementary material for: CBD2: A functional biomarker database for colorectal cancer
Source: Imeta. 2023 Dec 4;3(1):e155. doi: 10.1002/imt2.155 (PMC10989088; doi:10.1002/imt2.155)
Supplement: Supplementary file 1 — Supporting Information [file IMT2-3-e155-s004.docx]

**Supporting information to:**

**CBD2: a functional biomarker database for colorectal cancer**

**Running title**: CBD2: a functional biomarker database for colorectal cancer

Xueli Zhang^1,2,3,4#^; Min Li^1,5#^; Siting Ye^6,7#^; Ke Shen^8#^; Haining Yuan^9^; Shoaib Bakhtyar^10^; Qiliang Peng^11^; Yongsheng Liu^12^; Yingying Wang^13^; Manshi Li^13^; Chi Zhang^14^; Yixin Wang^5,15^; Xiaohe Bai^16^; Shunming Liu^3^; Ke Zhao^1,2,4,17^; Bairong Shen^18^; Dirk Repsilber^10^; Guang Hu^5*^; Hong Zhang^10*^; Xiao-Feng Sun^2*^

## AFFILIATIONS

^1^Medical Research Institute, Guangdong Provincial People’s Hospital (Guangdong Academy of Medical Sciences), Southern Medical University, Guangzhou, China

^2^Department of Oncology and Department of Biomedical and Clinical Sciences, Linköping University, Linköping, Sweden

^3^Guangdong Eye Institute, Department of Ophthalmology, Guangdong Provincial People’s Hospital (Guangdong Academy of Medical Sciences), Southern Medical University, Guangzhou, China

^4^Guangdong Provincial Key Laboratory of Artificial Intelligence in Medical Image Analysis and Application, Guangzhou, China

^5^MOE Key Laboratory of Geriatric Diseases and Immunology, Suzhou Key Laboratory of Pathogen Bioscience and Anti-infective Medicine, Center for Systems Biology, Department of Bioinformatics, School of Biology and Basic Medical Sciences, Suzhou Medical College of Soochow University, Suzhou, China

^6^Department of Ultrasound, The Second Affiliated Hospital of Guangzhou University of Chinese Medicine, Guangzhou, China

^7^Department of Orthopaedics, The Second Affiliated Hospital of Guangzhou University of Chinese Medicine, Guangzhou, China

^8^Department of Critical Care Medicine and Institutes for Systems Genetics, Frontiers Science Center for Disease related Molecular Network, West China Hospital, Sichuan University, Chengdu, China

^9^School of Laboratory Medicine and Bioengineering, Hangzhou Medical College, Hangzhou, China

^10^Institute of Medical Sciences, School of Medicine, Örebro University, Örebro, Sweden

^11^Department of Radiotherapy & Oncology, The Second Affiliated Hospital of Soochow University, Suzhou, China

^12^Department of Immunology, Genetics and Pathology, Uppsala University, Uppsala, Sweden

^13^Key Laboratory of Public Health Safety, School of Public Health, Fudan University, Shanghai, China

^14^Department of Otolaryngology, Guangzhou Women and Children’s Medical Centre, Guangzhou, China

^15^School of Medicine, The Chinese University of Hong Kong, Shenzhen, Shenzhen, China

^16^Department of Mathematics, University of California, San Diego, CA, U.S.A.

^17^Department of Radiology, Guangdong Provincial People’s Hospital, Guangdong Academy of Medical Sciences, Guangzhou, China

^18^Institutes for Systems Genetics, Frontiers Science Center for Disease-Related Molecular Network, West China Hospital, Sichuan University, Chengdu, China

# These authors contributed equally to this work.

## CORRESPONDENCE

*Prof. Xiao-Feng Sun, MD, PhD. E-mail: xiao-feng.sun@liu.se

*Prof. Hong Zhang, PhD. E-mail: hong.zhang@oru.se

*Prof. Guang Hu, PhD. E-mail: huguang@suda.edu.cn

**Figure S1. BBI networks for the existing diagnostic, therapeutic, and prognostic protein biomarkers in CBD2.** (A) BBI network of protein biomarkers for CRC diagnosis. (B) BBI network of protein biomarkers for CRC treatment. (C) BBI network of protein biomarkers for CRC prognosis.

**Figure S2. Functional analysis results for biomarkers included in CBD2.** Each section displays the top 10 most enriched Gene Ontology (GO) terms (Biological Process, Cellular Component, and Molecular Function) and the top 20 most enriched KEGG pathways. (A) Results for Diagnostic Biomarkers. (B) Results for Therapeutic Biomarkers. (C) Results for Prognostic Biomarkers.

**Table S1. The meaning of the parameters used to extract protein-protein interactions from the STRING database.**

**Table S2. Gene-to-miRNA Interaction Table.** This table illustrates the interactions between the provided list of genes and their associated miRNAs. Total interactions: 713 genes interacting with 72 distinct miRNAs.

**Table S3. miRNA-to-Gene Interaction Table.** This table illustrates the interactions between the provided list of miRNAs and their associated target genes. Total interactions: 26 miRNAs targeting 5887 distinct genes.

**Table S4. Results of KEGG enrichment analysis on the miRNA-gene network.** This table presents the results of the KEGG enrichment analysis performed on the constructed miRNA-gene network. Among the results, “Colorectal cancer” is ranked 6th.

**Table S5. Results of DisGeNET enrichment analysis on the miRNA-gene network.** This table presents the results of the DisGeNET enrichment analysis performed on the constructed miRNA-gene network. Among the results, “Colonic Neoplasms” is ranked 13th.
