# Supplementary material for: CBD2: A functional biomarker database for colorectal cancer
Source: Imeta. 2023 Dec 4;3(1):e155. doi: 10.1002/imt2.155 (PMC10989088; doi:10.1002/imt2.155)
Supplement: Supplementary file 3 — Figure S1: BBI networks for the existing diagnostic, therapeutic, and prognostic protein biomarkers in CBD2. Figure S2: Functional analysis results for biomarkers included in CBD2. [file IMT2-3-e155-s002.pdf]

How to Use

How to Search

We offer users three different ways to retrieve data: Categorical Search, Keyword Search, and Advanced Search. Each search strategy returns results with detailed information about the corresponding biomarker.

Categorical Search

Users can access biomarker data through the menu on the left side of the "Biomarkers" page. There are currently four major categories: Protein, RNA, DNA and Other, and clicking on a menu item will display a list of all the corresponding biomarkers on the right side. If there is a subcategory under a major category, the relevant submenu will appear when the user clicks on the menu item. Once the user clicks on one of the markers in the biomarker list, the interface will jump to the information page. (Figure 1)

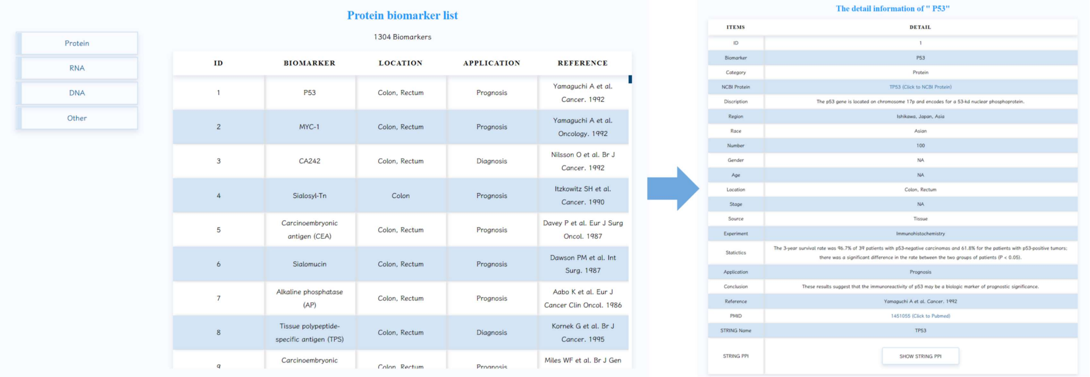

Figure 1. Search by category

Keyword Search

The second way to search the users needed marker is the key word search: input a biomarker name and then click the “search” button. It will turn to a result page including the relevant biomarker’s information. (Figure 2)

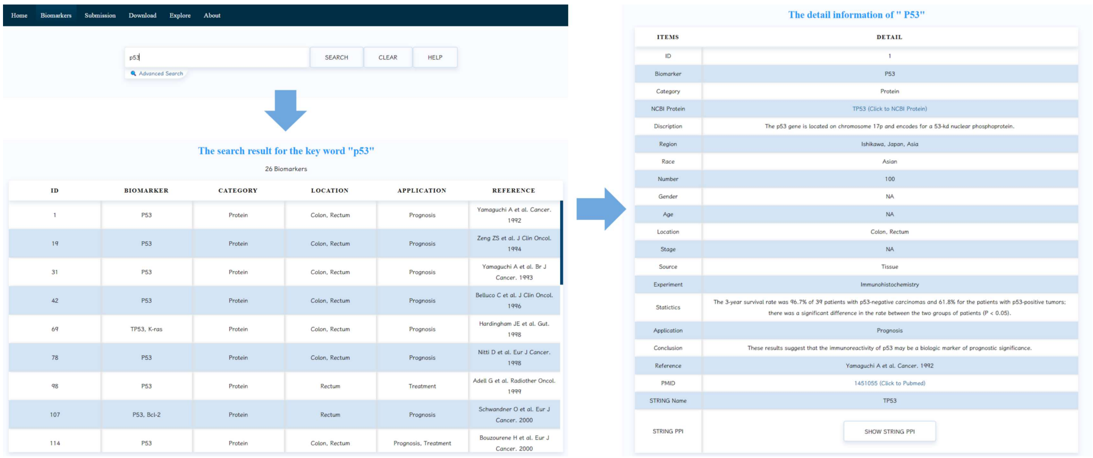

Figure 2. Search by key word

Advanced Search

Besides upon two ways, our database also provides the advanced search function: after click the "Advanced Search" button, an advanced search interface will appear. Six keywords are currently supported including region of study (Region), cancer stage (Stage), major cancer area (Location), marker source (Source), marker usage (Application), and information of published literature (Reference). (Figure 3)

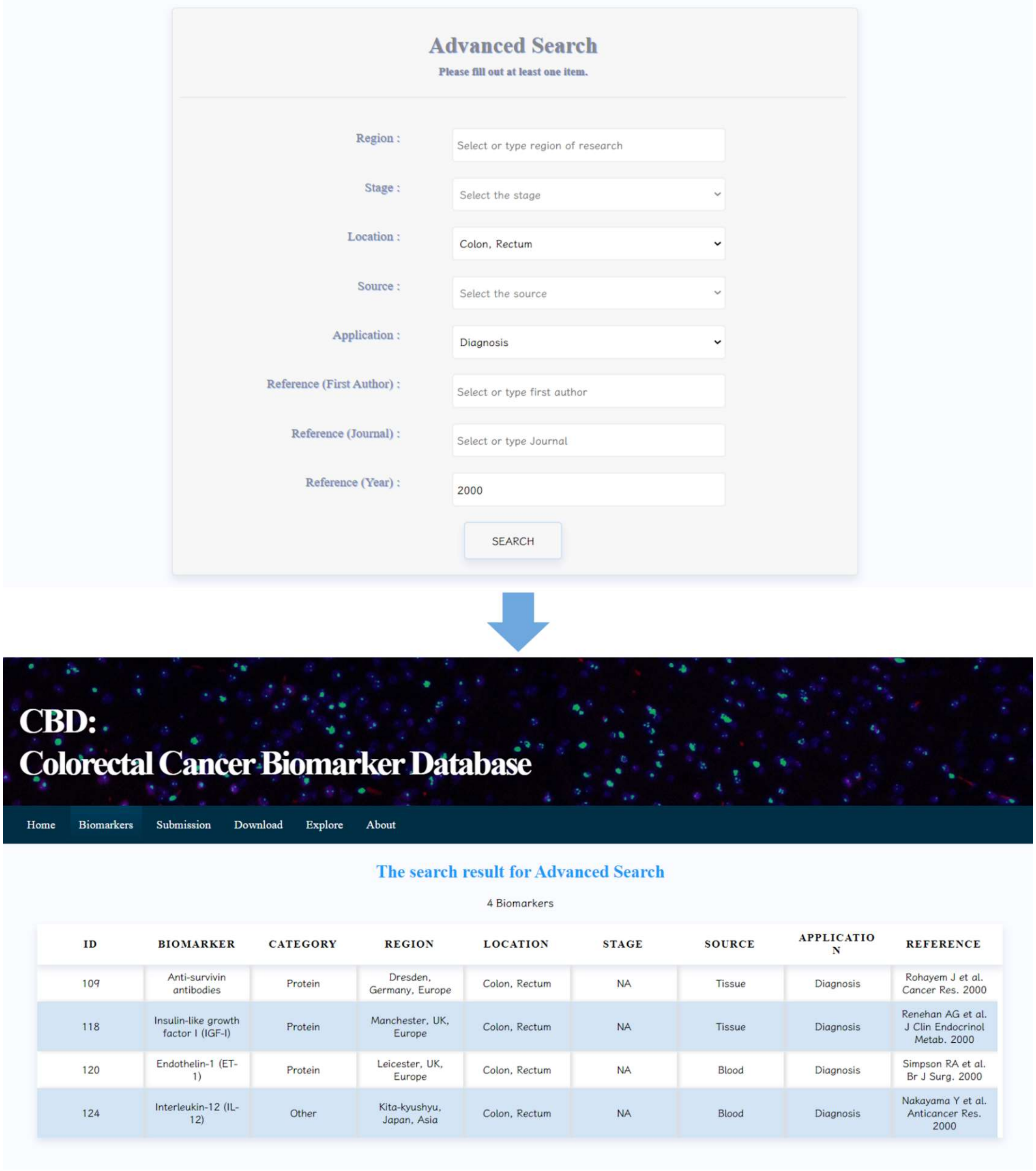

ID	BIOMARKER	CATEGORY	REGION	LOCATION	STAGE	SOURCE	APPLICATION	REFERENCE
109	Anti-survivin antibodies	Protein	Dresden, Germany, Europe	Colon, Rectum	NA	Tissue	Diagnosis	Rohayem J et al. Cancer Res. 2000
118	Insulin-like growth factor I (IGF-I)	Protein	Manchester, UK, Europe	Colon, Rectum	NA	Tissue	Diagnosis	Renehan AG et al. J Clin Endocrinol Metab. 2000
120	Endothelin-1 (ET-1)	Protein	Leicester, UK, Europe	Colon, Rectum	NA	Blood	Diagnosis	Simpson RA et al. Br J Surg. 2000
124	Interleukin-12 (IL-12)	Other	Kita-kyushyu, Japan, Asia	Colon, Rectum	NA	Blood	Diagnosis	Nakayama Y et al. Anticancer Res. 2000

Figure 3. Advanced Search

How to Explore

Network Display

Figure 4 below is our "Explore" page.

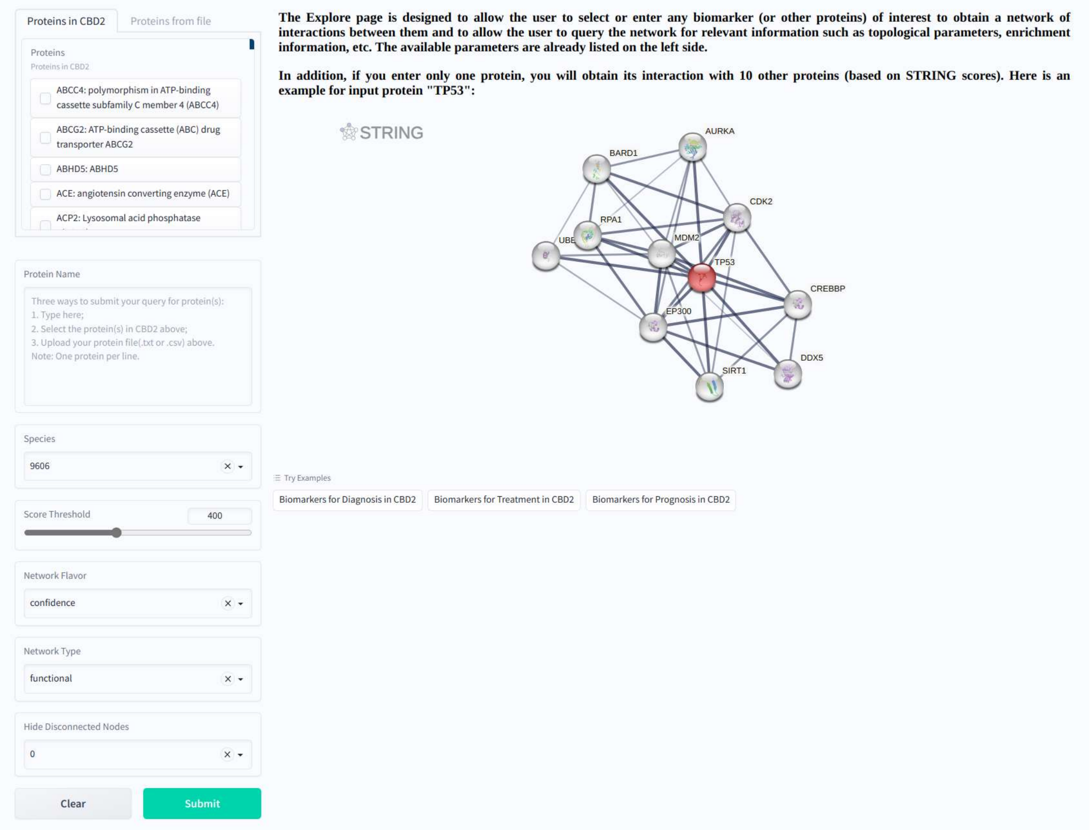

Figure 4. Explore page

The left area of the page provides biomarker input and parameter selection. Users can select or input biomarkers of interest in three ways: 1) directly select the biomarkers stored in CBD2 2) upload txt or csv files 3) Type directly in the text box. After clicking the "Submit" button, the resulting network will be displayed on the upper right. Note that if only one protein is queried, the results will by default show the 10 other proteins whose network interacts with the highest confidence, we have set the default query to TP53. (Figure 5)

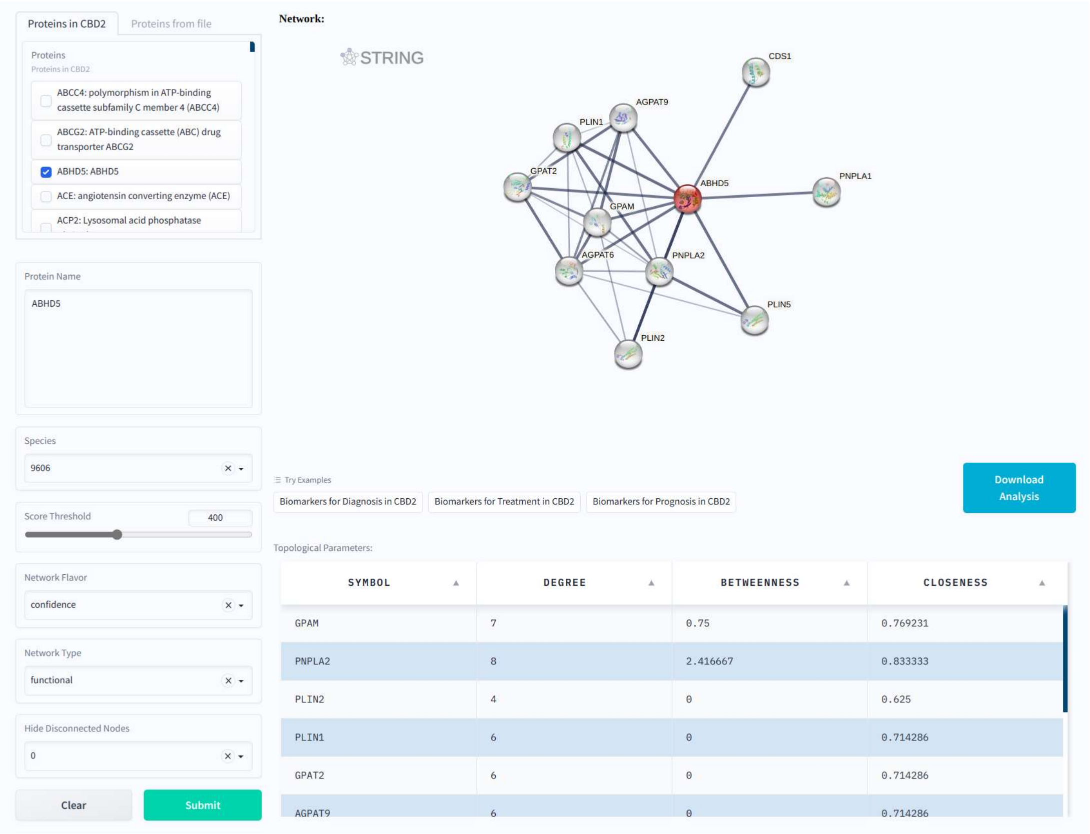

Figure 5. Network display

Network Analysis

At the same time, after the user clicks the "Submit" button, we will also return the list of network parameters and the list of enrichment analysis. By clicking the result entry id of the enrichment analysis, the user can jump to the result page of the corresponding database. (Figure 6)

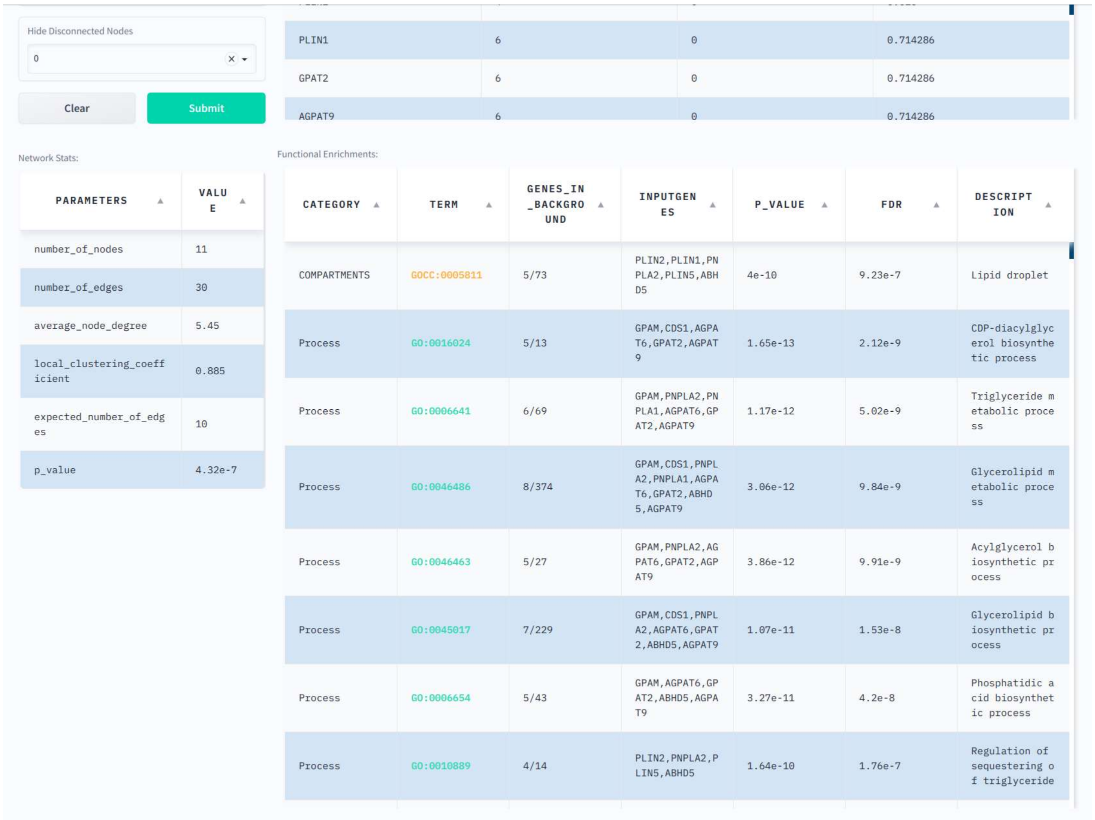

Hide Disconnected Nodes

0

Clear

Submit

PLIN1	6	0	0.714286
GPAT2	6	0	0.714286
AGPAT9	6	0	0.714286

Network Stats:

PARAMETERS	VALUE
number_of_nodes	11
number_of_edges	30
average_node_degree	5.45
local_clustering_coefficient	0.885
expected_number_of_edges	10
p_value	4.32e-7

Functional Enrichments:

CATEGORY	TERM	GENES_IN_BACKGROUND	INPUTGENES	P_VALUE	FDR	DESCRIPTION
COMPARTMENTS	GOCC:0005811	5/73	PLIN2,PLIN1,PNPLA2,PLIN5,ABHD5	4e-10	9.23e-7	Lipid droplet
Process	GO:0016024	5/13	GPAM,CDS1,AGPAT6,GPAT2,AGPAT9	1.65e-13	2.12e-9	CDP-diacylglycerol biosynthetic process
Process	GO:0006641	6/69	GPAM,PNPLA2,PNPLA1,AGPAT6,GPAT2,AGPAT9	1.17e-12	5.02e-9	Triglyceride metabolic process
Process	GO:0046486	8/374	GPAM,CDS1,PNPLA2,PNPLA1,AGPAT6,GPAT2,ABHD5,AGPAT9	3.06e-12	9.84e-9	Glycerolipid metabolic process
Process	GO:0046463	5/27	GPAM,PNPLA2,AGPAT6,GPAT2,AGPAT9	3.86e-12	9.91e-9	Acylglycerol biosynthetic process
Process	GO:0045017	7/229	GPAM,CDS1,PNPLA2,AGPAT6,GPAT2,ABHD5,AGPAT9	1.07e-11	1.53e-8	Glycerolipid biosynthetic process
Process	GO:0006654	5/43	GPAM,AGPAT6,GPAT2,ABHD5,AGPAT9	3.27e-11	4.2e-8	Phosphatidic acid biosynthetic process
Process	GO:0010889	4/14	PLIN2,PNPLA2,PLIN5,ABHD5	1.64e-10	1.76e-7	Regulation of sequestering of triglyceride

Figure 5. Network analysis

Download

The network image and network parameters could be downloaded by clicking the "Download" button.

PREVIOUS
Items in CBD

NEXT
FAQ

Copyright © 2018-2022 Colorectal Cancer Biomarker Database (CBD), Linköping University, Soochow University, Guangdong Provincial People's Hospital.
All Rights Reserved. Published with docsify.
